# Accuracy of patient‐specific instrumentation for implant positioning in custom‐made total ankle arthroplasty

**DOI:** 10.1002/jeo2.12026

**Published:** 2024-07-26

**Authors:** Antonio Mazzotti, Simone Ottavio Zielli, Alberto Arceri, Elena Artioli, Laura Langone, Federico Sgubbi, Giuseppe Geraci, Cesare Faldini

**Affiliations:** ^1^ IRCCS Istituto Ortopedico Rizzoli 1st Orthopaedics and Traumatologic Clinic Bologna Italy; ^2^ Department of Biomedical and Neuromotor Sciences (DIBINEM) Alma Mater Studiorum University of Bologna Bologna Italy

**Keywords:** patient‐specific instrumentation, preoperative planning, radiological analysis, tibial anterior surface angle, tibial lateral surface angle

## Abstract

**Purpose:**

This retrospective radiological analysis aimed to assess the accuracy of implant positioning in patients with ankle arthritis undergoing custom‐made total ankle arthroplasty (TAA) with patient‐specific instrumentation (PSI) compared with preoperative planning.

**Methods:**

Patients who underwent custom‐made TAA with PSI from January 2018 to March 2023 were retrospectively evaluated, focusing on the tibial anterior surface (TAS) angle, tibial lateral surface (TLS) angle and tibiotalar ratio (TTR). Additionally, data regarding the time from the preoperative computed tomography (CT) scan to surgery, associated procedures and complications were recorded.

**Results:**

No associated procedures were performed, and only one intraoperative complication, an iatrogenic lateral malleolar fracture, was recorded. In the coronal plane, custom‐made TAA with PSI consistently achieved precise positioning of prosthetic components, even in cases with significant preoperative deformities or bone deficits. However, a statistically significant deviation from the planned values was observed in the sagittal plane (*p* = 0.007). A notable correlation was identified between the time elapsed from the preoperative CT scan to surgery and the deviation from the planned to the actual postoperative TAS angle (*p* < 0.001).

**Conclusion:**

This study underscores the efficacy of PSI systems in achieving precise positioning in the coronal plane, in accordance with preoperative planning. In contrast, sagittal plane positioning did not demonstrate the same level of accuracy, as evidenced by a statistically significant difference between the planned and postoperative TLS values. Nevertheless, all measurements remained within the recommended range according to the existing literature.

**Level of Evidence:**

Level IV.

**Abbreviations**
AA: ankle arthritis
PSI: patient‐specific instrumentation
TAA: total ankle arthroplasty
TAS: tibial anterior surface
TLS: tibial lateral surface
TTR: tibiotalar ratio

## BACKGROUND

Total ankle arthroplasty (TAA) is a well‐established surgical option for treating end‐stage ankle arthritis (AA) [1]. The increasing adoption of TAA reflects advancements in implant designs and surgical techniques. Nevertheless, TAA outcomes have generally demonstrated lower success rates compared with hip or knee procedures, and the causes of such variations in results remain under investigation [4].

Accurate implant positioning and alignment are fundamental for the success of TAA, as even minor misalignments can significantly affect motion and contact pressure, potentially leading to postoperative residual pain and failure [23, 24].

Current TAA instrumentation may not consistently and adequately account for the patient's unique anatomical features: as a matter of fact, traditional systems typically rely on anatomical landmarks for reference points during surgery [5, 21, 25]. While experienced surgeons can achieve favourable implant positioning using standard techniques, natural gait and ankle motion may not be fully restored [5].

To enhance the prosthetic components positioning and better adhere to joint biomechanics, two innovative approaches have been introduced in TAA: patient‐specific instrumentation (PSI) and custom‐made implants.

PSI relies on personalised cutting guides tailored to each patient's unique anatomy based on preoperative ankle computed tomography (CT) scans, enhancing the precision of bone resection and implant positioning. PSI aims to enhance implant alignment, streamline operating room procedures, reduce costs and theoretically improve patient outcomes [17, 21].

Custom‐made implants have been developed as valid options for patients with insufficient bone stock for traditional TAA, particularly those seeking to preserve ankle mobility, with various models and materials [9, 10, 17]. Technological progress and the reduction in production costs have enabled an increasingly widespread use of custom implants [21].

While custom‐made implants have demonstrated distinct advantages, expanding the surgical indications to cases previously considered unsuitable for TAA, there is currently no consensus in the literature regarding the superiority of PSI, particularly in terms of component positioning [8, 13].

Most studies have focused on assessing the ability of preoperative planning to achieve a well‐aligned prosthesis on postoperative radiographs [26], without evaluating the correspondence between the planned and actual outcomes. To the best of our knowledge, there are currently no published reports assessing whether PSI ensures adherence to the positioning of custom‐made TAA prosthetic components according to the preoperative plan. This assessment gains even more significance considering the very nature of custom implants, which demand the utmost precision concerning preoperative planning.

Aim of this retrospective radiological analysis was therefore to evaluate the accuracy of implant positioning on the first postoperative weight‐bearing X‐rays in patients with AA who underwent custom‐made TAA using PSI in comparison to the preoperative planning.

## METHODS

This retrospective study adhered to the Declaration of Helsinki and received approval from our University Institutional Review Board.

Inclusion criteria encompassed a consecutive series of patients who underwent custom‐made TAA with PSI from January 2018 to March 2023 at our institution.

### Preoperative planning

Preoperative planning for custom‐made TAA using PSI involved obtaining preoperative ankle CT imaging following manufacturer‐established protocols to create a patient‐specific three‐dimensional (3D) model. For computer navigation systems, CT scans from the knee, including the anterior tibial tuberosity, through the mid‐foot, were required. The acquisition protocol entailed a spiral scan with the ankle positioned at a 90° dorsiflexion angle (kV: 120, mA: 60), employing a time rotation of 0.4–1 s. The field of view was set to encompass bone tissue, with a slice thickness of 0.6 mm and an overlapping slice interval.

CT scans enabled the identification of the mechanical axis of the tibia, running from the centre of the two tibial tuberosities to the centre of the tibiotalar joint. This axis is used to determine the preoperative rotational, coronal and sagittal plane deformity.

The implant positioning aimed to achieve, in relation to the mechanical axis of the tibia, a neutral aligned prosthesis in the coronal plane, with minimal forward orientation of the tibial component on the sagittal axis (87 ± 3°), as recommended by the literature [2, 32].

The selection of implant size was determined by the anatomy of the joint to be replaced. Custom‐made implants were specifically designed to both fill the bone defect and provide adequate bone support. The design process for tibial and talar implants utilised GeoMagic Wrap, combining anatomical surfaces from both the affected and contralateral tibiotalar joints, as previously outlined in the literature [9].

Once implant geometry and positioning were defined, corresponding PSIs were modelled on patients’ bone surfaces using GeoMagic Control. This process included bone resections and PSI design to match the frontal bone of the ankle, including osteophytes and irregularities of the tibial cortex, while also incorporating all necessary bone preparation guides.

The manufacturing phase began upon project approval. The personalised cutting guides used during surgery were made from biocompatible polyamide (PA12). Surgeons had access to sterile 3D‐printed plastic test models of implants and PSIs before and during surgery. Custom‐made tibial and talar components were constructed with both full‐scale models and a scaled‐down model, 10% smaller than CT measurements.

The time elapsed between the performance of the CT scan, which was used for subsequent surgical planning, and the surgical procedure itself was recorded.

### Surgical technique

All the procedures were performed by two experienced foot and ankle orthopaedic surgeons using a standard technique and a three‐component ankle prosthesis (FAR ankle prosthesis—Adler Ortho ©). The patient was placed in a supine position, and a tourniquet was applied at the root of the thigh. Surgery was carried out through an anterior approach, exploiting the gap between the tibialis anterior tendon and the extensor digitorum longus tendon. Particular attention was paid to preserve the anterior tibial osteophytes, crucial for achieving accurate PSI alignment, using an electric saw.

Personalised tibial and talar cutting guides were employed for resections. In instances requiring total talar replacement, which involved a complete talectomy, the preparation of the subtalar joint and the drilling holes for the calcaneal screws was carried out through dedicated perforation guides. Subsequently, the definitive talar and tibial components, in addition to the meniscus, were inserted. Implant movement and stability were clinically tested, and fluoroscopic control confirmed proper positioning.

Following surgery, a plaster cast was applied for 3 weeks, with no weight bearing allowed. After cast removal, progressive weight bearing on a walking boot was permitted, and ankle mobilisation was encouraged. In cases of additional procedures or intraoperative complications, full weight bearing was postponed for at least 30 days, in accordance with radiographic findings.

### Outcomes collection

Preoperative positioning of the implant components was based on a two‐dimensional reconstruction of the preoperative planning in the anteroposterior (AP) and lateral views.

Postoperative implant positioning was evaluated using the AP and lateral radiographs at the first postoperative weight‐bearing radiographs, which can be considered the most accurate representation of the intraoperative prosthesis placement.

**Figure 1:**
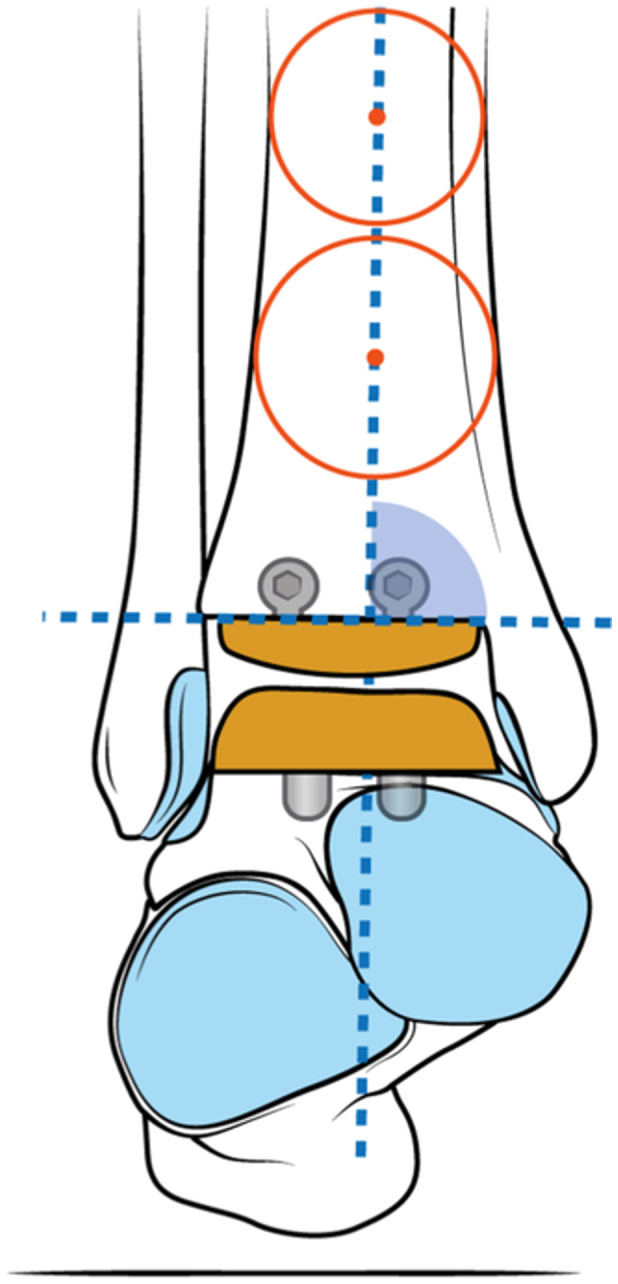
Tibial anterior surface (TAS) angle. Two circles were drawn (red), fitting between the medial/lateral tibial cortices 5 and 10 cm above the joint line. Then, a line (blue) was drawn to connect the centres of both circles, extending distally to the most distal point of the component, thus providing the mechanical axis of the tibia. The medial‐superior angle formed by the intersection of the mechanical axis of the distal tibia and the flat portion of the tibial component was identified as the tibial anterior surface (TAS) angle.

A standardised method was used to calculate the mechanical axis of the distal tibia in both AP and lateral planes [20, 30, 32]. This method involved drawing two circles, with one fitting between the medial and lateral cortices at the proximal part of the tibial shaft and the other fitting between the medial and lateral metaphyseal flares and the distal extent of the component. A line was drawn to connect the centres of both circles, extending distally to the most distal point of the component, thus providing the mechanical axis of the tibia (Figure 1).

In the AP view, the angle formed by the intersection of the mechanical axis of the distal tibia and the flat portion of the tibial component was identified as the tibial anterior surface (TAS) angle [27] (Figure 1).

**Figure 2:**
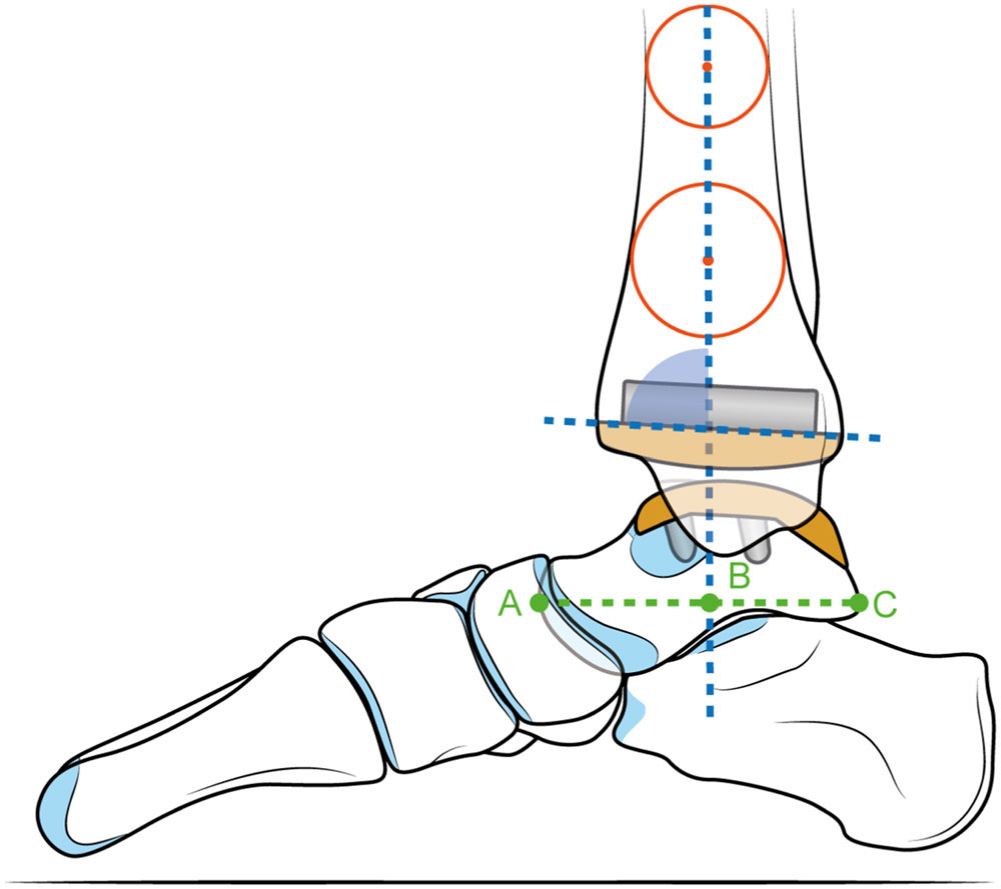
Tibial lateral surface (TLS) angle and tibiotalar ratio (TTR). Two circles were drawn (red), fitting between the antero/posterior tibial cortices 5 and 10 cm above the joint line. Then a line (blue) was drawn to connect the centres of both circles, extending distally to the most distal point of the component, thus providing the mechanical axis of the tibia. The tibial lateral surface (TLS) angle is defined as the intersection of the distal tibial mechanical axis and the flat portion of the tibial component. The tibiotalar ratio (TTR) is the ratio between the length of the posterior segment of the talus (green BC segment) to the total longitudinal talar length (green AC segment) and measures the anterior or posterior subluxation of the talus.

In the lateral view, two measures were detected: the tibial lateral surface (TLS) angle and the tibiotalar ratio (TTR). TLS angle is defined as the intersection of the distal tibial mechanical axis and the flat portion of the tibial component [7] (Figure 2), while TTR is the ratio between the length of the posterior segment of the talus to the total longitudinal talar length, and measures the anterior or posterior subluxation of the talus [29] (Figure 2).

These angles were measured using a picture archiving and communication system (PACS) equipped with RadiAnt DICOM Viewer (version 2021.1.).

All measurements were performed independently by three foot and ankle orthopaedic surgeons with extensive experience in performing radiographic analysis. Measurements were performed twice in separate sessions. The measurements considered for statistical analysis were the result of averaging the measurements taken by the three researchers for each assessment. Accuracy referred to how closely the implant positioning matched the preoperative planning [15].

Furthermore, all intraoperative complications were collected.

### Statistical analysis

Intraobserver reliability was assessed using intraclass correlation coefficients (ICCs) for continuous data, with a Cronbach's *⍺* exceeding 0.80 signifying perfect reliability. Cohen's *κ* coefficient was calculated to measure interobserver reliability. Variables resulting from clinical scores were reported as mean values, standard deviations (SDs) and ranges. Data distribution of continuous variables was assessed for normality using the Shapiro–Wilk test. Following confirmation of normal distribution, paired *t* tests were conducted to compare preoperative and final follow‐up clinical scores. Any potential correlation between two variables was assessed using the Pearson's test. Statistical significance was defined as *p* < 0.05. Statistical analyses were performed using Jamovi software (The Jamovi project—jamovi Version 1.6, 2021).

## RESULTS

Fourteen patients underwent TAA surgery with PSI. Among these, nine were male, averaging 52.4 ± 10.1 years old at the time of the operation.

All patients had a posttraumatic aetiology, and 21.4% of them (three out of 14) had a history of primary TAA failure. Two of these patients experienced septic mobilisation, necessitating a multistep therapy that included employing a cemented spacer and targeted antibiotic treatment until imaging and laboratory parameters normalised.

The average time elapsed between the preoperative CT scan and the surgical procedure was 7.79 ± 3.19 weeks, with a range of 6–18 weeks.

**Figure 3:**
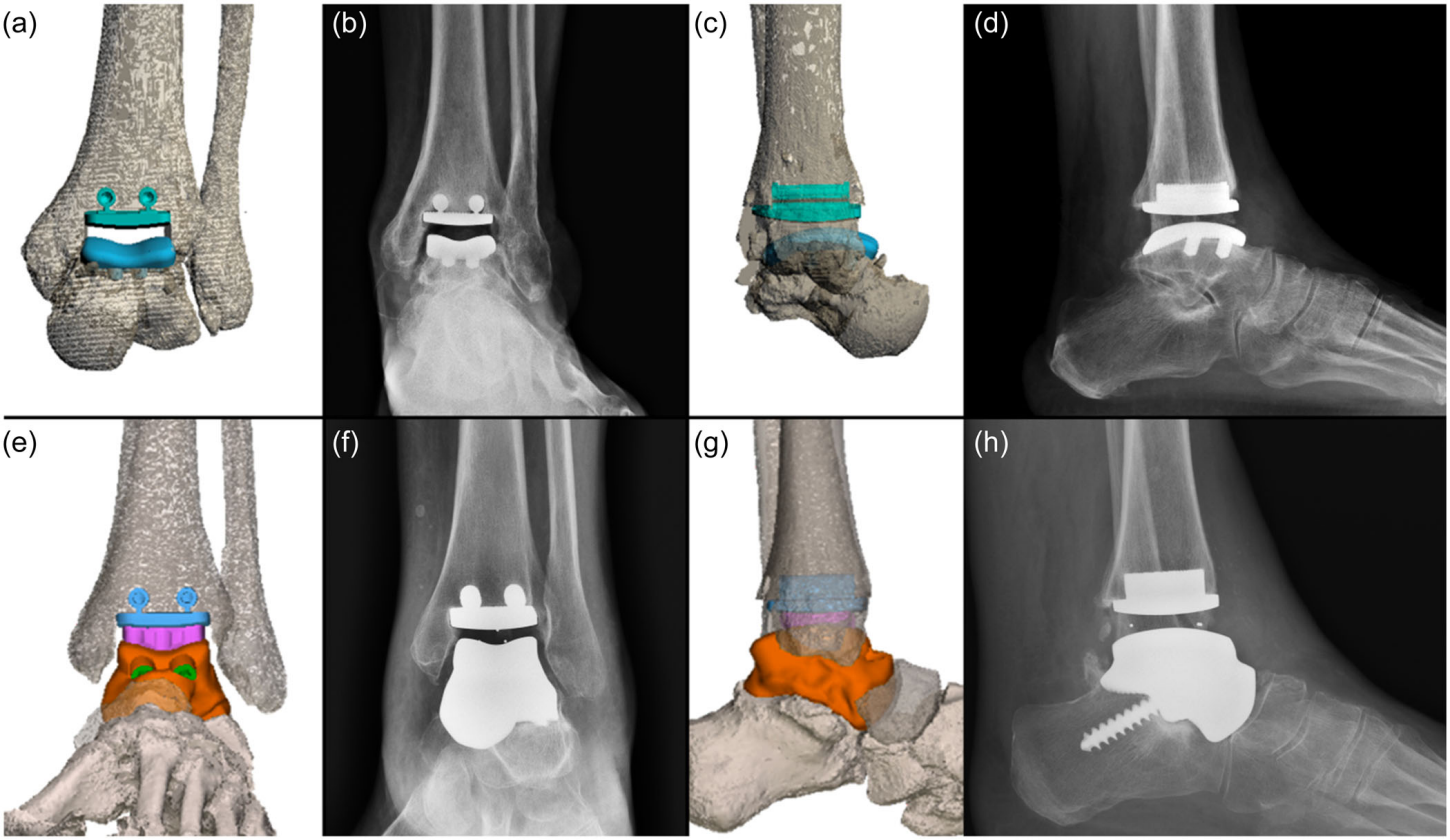
Two cases of patients with their respective preoperative planning and postoperative X‐rays. (a) Preoperative antero‐posterior view planning of patient 2; (b) postoperative antero‐posterior view weight‐bearing x‐rays of patient 2; (c) preoperative lateral view planning of patient 2; (d) postoperative lateral‐view weight‐bearing X‐rays of patient 2; (e) preoperative antero‐posterior view planning of patient 13; (f) postoperative antero‐posterior view weight‐bearing X‐rays of patient 13; (g) preoperative lateral view planning of patient 13; (h) postoperative lateral‐view weight‐bearing X‐rays of patient 13.

The characteristics of the custom implants utilised are detailed in Table 1, while examples of preoperative planning and postoperative weight‐bearing X‐rays are presented in Figure 3.

**Table 1:** Patients’ demographic and implants characteristics.

Patient	Age at the surgery (years)	Time from preop CT to surgical procedure (weeks)	Implant characteristics	
			Tibia component	Talus component
1	56	7	Resurfacing–standard design	Resurfacing–standard design
2	57	6	Resurfacing–standard design	Resurfacing–standard design
3	50	18	Resurfacing–standard design	Total talus component
4	45	10	Resurfacing–standard design	Total talus component
5	30	6	Resurfacing–standard design	Total talus component
6	58	6	Augmented tibial component	Total talus component
7	42	9	Resurfacing–standard design	Resurfacing–standard design
8	48	6	Resurfacing–standard design	Resurfacing–standard design
9	49	7	Resurfacing–standard design	Resurfacing–standard design
10	69	7	Resurfacing–standard design	Augmented talus component
11	51	6	Resurfacing–standard design	Resurfacing–standard design
12	67	7	Resurfacing–standard design	Resurfacing–standard design
13	61	5	Resurfacing–standard design	Total talus component
14	51	6	Resurfacing–standard design	Total talus component
Mean	52.4 ± 10.1	7.79 ± 3.19		

Abbreviation: CT, computed tomography.

Preoperatively two patients exhibited a valgus deformity (up to 7.8°) and six had a varus deformity (up to 10.8°), with a total of six patients displaying neutral alignment ( ± 5°). Preoperative and postoperative radiological parameters are presented in Tables 2 and 3, respectively.

**Table 2:** Preoperative X‐ray parameters.

Patient	Preop TAS (°)	Varus (–) and valgus (+) deformity (°)	Absolute TAS deformity (°)	Preop TLS (°)	Preop TTR (ratio)
1	86	−4	4	81.8	50.6
2	87.1	−2.9	2.9	89	35.4
3	97.8	7.8	7.8	80.1	36.2
4	84	−6	6	87	38.8
5	84	−6	6	85	48.1
6	84.5	−5.5	5.5	84.4	30.6
7	88.1	−1.9	1.9	86.1	49.4
8	83.5	−6.5	6.5	83.7	62.4
9	84.9	−5.1	5.1	87.1	37.1
10	91.1	1.1	1.1	87.5	42.1
11	97.2	7.2	7.2	82.6	39.7
12	91.6	1.6	1.6	84.7	44.7
13	79.2	−10.8	10.8	74.6	61.4
14	91	1	1	76.64	37.8
Mean	87.88 ± 5.32	−2.14 ± 5.32	4.81 ± 2.88	83.59 ± 4.15	43.88 ± 9.53

Abbreviations: TAS, tibial anterior surface; TLS, tibial lateral surface; TTR, tibiotalar ratio.

**Table 3:** Postoperative X‐ray parameters.

Patient	Postop TAS (°)	Varus (–) and valgus (+) deformity (°)	Absolute TAS deformity (°)	Postop TLS (°)	Postop TTR (ratio)
1	87.97	−2.03	2.03	86.34	51.21
2	91.95	1.95	1.95	90.66	47.41
3	79.97	−10.03	10.03	82.29	60.67
4	88.7	−1.3	1.3	84.06	57.04
5	91.49	1.49	1.49	78.17	61
6	88.98	−1.02	1.02	83.26	43.56
7	85.41	−4.59	4.59	85.96	44.9
8	88.64	−1.36	1.36	85.06	37.67
9	87.98	−2.02	2.02	87.14	50.31
10	89.61	−0.39	0.39	90.11	37.58
11	90.37	0.37	0.37	87.84	30.79
12	87.53	−2.47	2.47	90.74	30.35
13	90	0	0	90.95	36.17
14	89.56	−0.44	0.44	83.25	34.22
Mean	87.13 ± 3.77	−1.56 ± 2.94	2.10 ± 2.55	88.44 ± 2.95	44.49 ± 10.51

Abbreviations: TAS, tibial anterior surface; TLS, tibial lateral surface; TTR, tibiotalar ratio.

Two patients required Achilles tendon lengthening, performed percutaneously using the Hoke technique [12].

**Figure 4:**
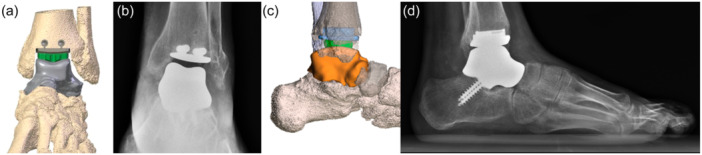
Preoperative planning and postoperative X‐rays of patient 3. (a) Preoperative antero‐posterior view planning of patient 3; (b) postoperative antero‐posterior view weight‐bearing X‐rays of patient 3; (c) preoperative lateral view planning of patient 3; (d) postoperative lateral‐view weight‐bearing X‐rays of patient 3.

One patient (n. 6) experienced intraoperative complications, an iatrogenic lateral malleolar fracture, which was synthesised through 2 K‐wires. Patient number 3 was the only one to require the smaller option for the custom‐made talus component (Figure 4).

After collecting the prosthetic component positioning parameters, intraobserver reliability demonstrated ICC values of 0.94, 0.96 and 0.93 for the three orthopaedic surgeons, respectively, while interobserver reliability showed a kappa coefficient of 0.93. Following the Shapiro–Wilk test, a normal distribution of the variables was observed. Planned and postoperative data of the analysed parameters are presented in Table 2.

TAS and TTR did not exhibit statistically significant differences, indicating a strong correspondence between preoperative planning and postoperative radiographic assessment (Table 4). The only statistically significant difference observed was between the planned and postoperative TLS values, with a *p* value of 0.007 (Table 4).

**Table 4:** Statistical analysis of planned and postoperative X‐ray parameters.

Outcomes	Planned values (°)	Postoperative values (°)	Δ values (°)	Statistical analysis
TAS	89.64 ± 1.21	88.44 ± 2.95	1.57 ± 2.28	*p* = 0.088
TLS	88.00 ± 2.11	86.13 ± 3.77	2.36 ± 1.61	*p* = 0.007*
TTR	45.07 ± 11.02	44.49 ± 10.51	13.45 ± 9.49	*p* = 0.900

Abbreviations: TAS, tibial anterior surface; TLS, tibial lateral surface; TTR, tibiotalar ratio.

* Statistically significative value.

**Figure 5:**
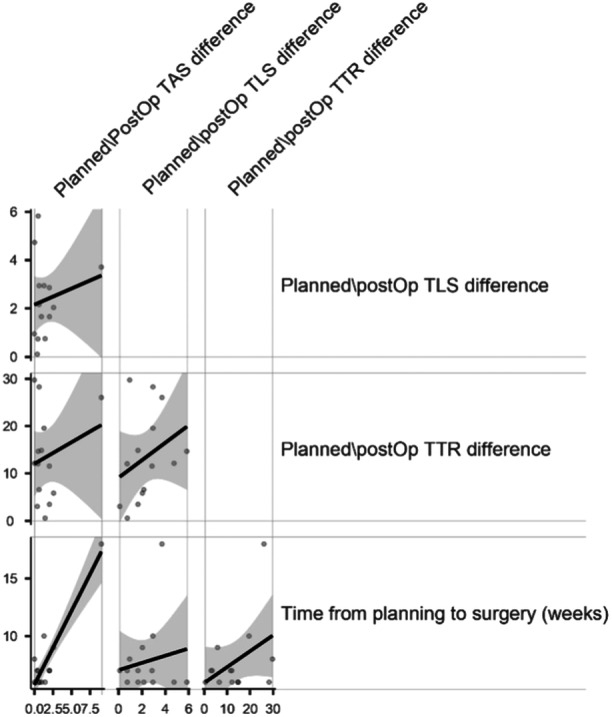
Graphic representation of the Pearson's correlation analysis.

Regarding correlation, a statistically significant relationship was observed between the time elapsed from the preoperative CT scan to the surgery and the disparity between the planned and actual postoperative TAS, with a *p* value of less than 0.001 (Figure 5).

## DISCUSSION

This study underscores the efficacy of PSI systems, even in scenarios involving significant coronal plane deformities or bone deficits, in achieving precise custom‐made TAA component positioning in accordance with preoperative planning.

Achieving mechanical alignment while addressing coronal plane deformities in TAA is essential to reduce complications such as eccentric wear, component loosening, subsidence and the need for reoperation [15, 18, 19]. As a matter of fact, a systematic review of 49 studies identified component loosening and/or subsidence as the primary reasons for TAA revision, accounting for 28% of cases [11].

Most authors have primarily focussed on evaluating preoperative planning's ability to achieve a well‐aligned prosthesis on postoperative radiographs, without assessing the correspondence between the planned and achieved results [6, 14, 28].

The few specific studies on this topic have been conducted on specimens. For example, in a cadaveric model, Berlet et al. [3] reported on the use of patient‐specific guides for TAA and found that surgical plans and guides consistently provided reliable and reproducible placement of TAA implants. The average variation between preoperative planning and actual postoperative implant placement was less than 2° and 1.4 mm in all tested specimens.

The importance of preoperative planning reproducibility in prosthetic component positioning becomes even more pronounced when dealing with custom‐made TAA. 3D‐printed prosthetic components need to conform to the patient's distinctive anatomy, often characterised by bone stock deficits, and are designed with submillimetric tolerances. Furthermore, the patient's pathological anatomy is often compromised, with challenges in using typical anatomical landmarks and frequent associations with supra‐ or infra‐articular deformities.

To date, there are no studies that examine the accuracy of implant positioning in patients with AA who have undergone custom‐made TAA with the use of PSI, comparing it to the preoperative planning.

The results of this case series demonstrate that even in cases with significant coronal and sagittal plane deformities, reaching up to 10.8° in the coronal plane, or severe bone stock deficits, such as total talar necrosis, PSI allows for precise positioning of prosthetic components, aligning with preoperative planning. Among similar in vivo studies investigating this parameter, Daigre et al. [6] reported a coronal alignment correction from an average preoperative value of 4.6° to a mean postoperative value of 1.8°. Nevertheless, preoperative coronal deformities ranged from 5° varus to 3.6° valgus, which are significantly lower values compared with those of the current study.

Hsu et al. published early clinical results using the Prophecy system, involving both Infinity and Inbone II TAA. Their case series exhibited preoperative coronal plane deformities ranging from 14° of valgus to 10° of varus. The reported average difference between planned and postoperative coronal alignment was within 3°, comparable to the results observed in this case series.

In a similar study by Thompson et al. [28], 100% of postoperative implant positioning in the coronal and sagittal planes fell within 5° of the preoperative template. However, preoperative deformities in this cohort were minimal, with an average preoperative coronal disalignment of 0.84°, ranging from 0.19° to 2.4°. It is also worth noting that the study by Thompson et al. included only 10 patients and involved the use of two different prosthetic models, one aligned with the anatomic axis and the other with the mechanical axis.

Notably, one patient (N. 3) in this study exhibited a postoperative TAS alignment significantly deviating from the planned value, measuring 10.03°. This discrepancy may be attributed to various factors. One possible explanation could be the imperfect adherence of the cutting guides to the bony surfaces. However, we took great care to collaborate with engineers during the design of the cutting guides, ensuring the preservation of anatomical landmarks, primarily the tibial osteophytes, of sufficient dimensions. This approach allowed for secure and consistent placement of the cutting guide in most instances. Another potential explanation could be a longer time gap between the preoperative CT scan and the surgery, leading to an imperfect correspondence between the patient's anatomy and the preoperative CT, resulting in PSI inaccuracy and a subsequent iatrogenic fracture. As previously suggested, in cases of customised TAA with PSI for patients with severe bone loss, minimising the time between the preoperative CT scan, planning and surgery is crucial [9]. It is also advisable to have a prosthetic component approximately 9/10 in scale compared with the contralateral side, as suggested by Hussain et al. [16], especially to address potential challenges associated with talar component positioning.

Regarding sagittal alignment, the postoperative TLS values exhibited an average difference of 2.37° compared with the planned values, which was statistically significant. Preoperatively, TLS values exceeded the physiological range (less than 83°) in five out of 14 patients (37%). The inferior accuracy concerning TLS may be attributed to the anterior surgical approach, which gives less awareness of sagittal plane alignment. Therefore, even small imperfections in the adherence of cutting guides to the bone surface can compromise accuracy. A similar case series by Daigre et al. [6] reported a postoperative average TLS of 87.6°, ranging from 83.5° to 95.0°, consistent with our findings.

TTR interpretation is complex, as no established reference values exist. According to Wood et al. [32], this value seems associated with the TLS. Specifically, when the tibia slopes forward (below 83°), the TTR tends to decrease, while a neutral slope (TLS between 83° and 90°) tends to normalise it. Therefore, analysing these two values together is advisable. In this study, no significant correlation between these values was observed.

The postoperative TLS values in this study align with the recommended range of 83–90° [2, 32], indicating a slight anterior orientation of the tibial prosthetic component. This inclination stems from the author's preference for a TLS angle below 90°, aimed at avoiding talus impingement during dorsiflexion, without reaching levels that could result in anterior talus subluxation.

Regarding the predictability of implant sizing through preoperative planning, only one case required a 90% scaled version of the predicted dimensions, corresponding to the patient who encountered an intraoperative fracture, resulting in a prediction rate of 92.86%. These data are consistent with findings in the literature concerning the tibial component, while indicating a superior prediction rate for the talar component, ranging from 46% to 90% [6, 14, 28].

One possible explanation for the talar component's deviation, as previously proposed by Daigre et al., is the variability in gutter debridement, which can range from aggressive to minimal depending on the surgeon's preferences. In this case series, the utilisation of cutting guides allowed for effective gutter debridement, obviating the need for additional recuts and thereby improving the accuracy of component size prediction. The sole observed discrepancy pertained to patient N. 3, who, as previously explained, experienced a delayed CT scan‐to‐surgery time interval. In this time frame, this patient continued to bear weight on the limb to be operated on, potentially exacerbating the ankle anatomy. In this specific case, once all the cuts at the tibia and tarsus levels were made, it was physically impossible to insert the component produced at 100% scale.

It is noteworthy that, to date, most preoperative planning systems primarily rely on the anatomic alignment of the tibial component. Even in cases where preoperative CT scans are conducted under load, they frequently provide bony information from the knee downwards, overlooking the patient's atypical position during these scans. Furthermore, to date, all the available PSI systems align the TAA in relation to the mechanical axis of the tibia. However, a nonnegligible percentage of patients with AA, due to posttraumatic sequelae, may exhibit lower limb deformities that do not adhere to the assumption that the anatomical axis of the tibia corresponds to the mechanical axis.

It should be noted that the actual utility of PSI is still under discussion, as some authors have not reported significantly higher accuracy compared with traditional systems [26]. In this context, the real utility of PSI may lie in its application in complex cases characterised by significant deformities in the coronal and sagittal planes, combined with a mechanical axis of the tibia that is not reliably predictable.

Limitations of the present study include the retrospective design and the relatively small patient cohort, which limited the statistical power of the study's results. Nonetheless, it should be noted that a significant proportion of patients treated with this technique would not have been suitable candidates for a standard ankle prosthesis due to the severe acquired bone deficits. The high complexity of the cases under investigation also limits the availability of large case series, as reflected in the existing literature [9, 22, 31]. Clinical or functional outcomes were not evaluated, given the radiographic focus of the study. Long‐term radiographic analysis of potential implant subsidence, osteolysis and impingement were not observed; however, longer follow‐up data are necessary to detect these potential complications. If the primary aim of this study was to evaluate the accuracy of PSI in replicating preoperative planning, it is important to underscore the absence of a control group treated with the current gold standard in preoperative prosthetic planning, namely, radiographic planning with landmarks. Nevertheless, obtaining a sample of patients with characteristics comparable to the examined group would have been challenging.

In addition to these considerations, it is noteworthy that all cases examined in this study exhibited no supra‐ or infra‐articular deformities. The authors firmly believe that achieving favourable outcomes with TAA necessitates the absence of significant concurrent deformities at the ankle or foot level. If such deformities are identified, addressing them concurrently or, preferably, prior to TAA through a two‐stage procedure is crucial.

## CONCLUSION

This study underscores the efficacy of PSI systems in achieving precise positioning in the coronal plane, in accordance with preoperative planning. In contrast, sagittal plane positioning did not demonstrate the same level of accuracy, as evidenced by a statistically significant difference between the planned and postoperative TLS values.

Nevertheless, all measurements remained within the recommended range according to the existing literature.

## AUTHOR CONTRIBUTIONS

Each author has contributed significantly to the study design, data acquisition and interpretation. In particular, Antonio Mazzotti, Simone Ottavio Zielli and Cesare Faldini brought valuable clinical insight, significantly contributing to the design and implementation of our study. Alberto Arceri, Elena Artioli, Laura Langone and Federico Sgubbi played a key role in the data collection phase, demonstrating extraordinary precision and dedication. Antonio Mazzotti, Cesare Faldini, Giuseppe Geraci and Simone Ottavio Zielli critically analysed the data, making a significant contribution to result interpretation and conclusion formulation. Cesare Faldini drafted and critically review the scientific manuscript. All authors have been actively involved in the drafting and critical revision of the manuscript, and all provided final approval of the version to be published.

## CONFLICT OF INTEREST STATEMENT

The authors declare no conflict of interest.

## ETHICS STATEMENT

This study received approval from our university institutional review board. Written informed consent was obtained from all patients included in this study.

## Data Availability

The data that support the findings of this study are available from the corresponding author upon reasonable request.
